# Dual Functional Recovery: The Contrasting Roles of Volunteering and Helping Behaviors

**DOI:** 10.1093/geroni/igaf122.207

**Published:** 2025-12-31

**Authors:** Samuel Nemeth, Seoyoun Kim, Cal Halvorsen, Shawn Bauldry

**Affiliations:** Purdue University, West Lafayette, Indiana, United States; University of Michigan, Ann Arbor, Michigan, United States; Washington University in St. Louis, St. Louis, Missouri, United States; Purdue University, West Lafayette, Indiana, United States

## Abstract

The concept of dual functionality integrates both physical and cognitive function – two important and interrelated domains for older adult health and well-being – and represents the absence of impairment in either domain. The loss of dual function presents a significant barrier to independent living; however, this loss may not be permanent. As such, guided by social integration theory, this study investigates the effect of two prosocial behaviors – formal volunteering and informal helping – on the recovery of dual function. We use nationally representative panel data from the Health and Retirement Study (2004-2016, N = 6,110), entropy balancing weights, and discrete time hazard models. After adjusting for selection and study covariates, volunteering was associated with lowered odds of recovery while helping was associated with increased odds of recovery (Odds Ratio (OR)=0.863 [95% CI: 0.754, 0.987]; OR = 1.120 [95% CI: 1.010, 1.243], respectively). Additionally, we found that age at loss of dual function moderated the effect of volunteering and helping (p < 0.05). Specifically, there is a significant difference in the effects of volunteering and helping when dual function is lost at younger ages. Findings underscore the complexity of recovery from impairment, the role prosocial behaviors may play in this process for older adults, and provide implications for health policy and interventions.

